# Feasibility Study of Extended-Gate-Type Silicon Nanowire Field-Effect Transistors for Neural Recording

**DOI:** 10.3390/s17040705

**Published:** 2017-03-28

**Authors:** Hongki Kang, Jee-Yeon Kim, Yang-Kyu Choi, Yoonkey Nam

**Affiliations:** 1 Department of Bio and Brain Engineering, Korea Advanced Institute of Science and Technology (KAIST), Daejeon 34141, Korea; hongki.kang@kaist.ac.kr; 2 Department of Electrical Engineering, Korea Advanced Institute of Science and Technology (KAIST), Daejeon 34141, Korea; jyeon@nobelab.kaist.ac.kr; 3 KAIST Institute for the NanoCentury, Korea Advanced Institute of Science and Technology (KAIST), Daejeon 34141, Korea

**Keywords:** silicon nanowire, field-effect transistor (FETs), neural recording, 1/f noise, random telegraph noise, microelectrode array

## Abstract

In this research, a high performance silicon nanowire field-effect transistor (transconductance as high as 34 µS and sensitivity as 84 nS/mV) is extensively studied and directly compared with planar passive microelectrode arrays for neural recording application. Electrical and electrochemical characteristics are carefully characterized in a very well-controlled manner. We especially focused on the signal amplification capability and intrinsic noise of the transistors. A neural recording system using both silicon nanowire field-effect transistor-based active-type microelectrode array and platinum black microelectrode-based passive-type microelectrode array are implemented and compared. An artificial neural spike signal is supplied as input to both arrays through a buffer solution and recorded simultaneously. Recorded signal intensity by the silicon nanowire transistor was precisely determined by an electrical characteristic of the transistor, transconductance. Signal-to-noise ratio was found to be strongly dependent upon the intrinsic 1/f noise of the silicon nanowire transistor. We found how signal strength is determined and how intrinsic noise of the transistor determines signal-to-noise ratio of the recorded neural signals. This study provides in-depth understanding of the overall neural recording mechanism using silicon nanowire transistors and solid design guideline for further improvement and development.

## 1. Introduction

Neural recording technique is one of the most vital tools to monitor and modulate brain activities. Access to the brain activities is required for implementing brain–machine interface applications. For the non-invasive measurement of neural signals simultaneously from multiple cells, extracellular neural recording has been performed using microelectrode arrays (MEAs) with the recording electrode sizes in the range of 20–60 µm in diameter when the typical size of a mammalian neuron is 10–20 µm in diameter. There are broadly two types of MEAs [1,2]: (1) passive MEAs with passive electrodes interfacing with neuron cells, which are connected to external amplifier systems for further signal processing [3]; (2) active MEAs with active electronic components such as field-effect transistors (FETs) or integrated circuits directly interfacing with the cells and amplifying the cellular signals [4]. While passive MEAs are more dominantly used (e.g., planar passive MEAs) for the extracellular neural recording, active MEAs have been known to offer unique benefits. First of all, since the neural signal is recorded right at the cell–electrode interface, on-chip amplification and filtering with minimized parasitics and interferences would allow more reliable data recording and processing. Second, much higher electrode density is achievable by the integration of multiplexer for the multichannel signals [5]. Third, the compact design of the entire recording system and its small physical area would be especially beneficial for implanted in vivo applications [6]. Last, the capability of on-chip logic implementation enables additional functionalities such as cyclic voltammetry, pH or temperature sensors [7].

Among various types of the active MEAs, nanowire field-effect transistor (NW FET) has been of particular interests after the demonstration of its significantly increased recorded signal strength [8,9,10]. Millivolt (mV)-range signals were recorded by the NW FETs whereas the neural signal recorded by planar passive MEAs is only in the range of hundreds of µV. It was postulated that high surface-to-volume ratio of the nanostructures is particularly helpful for enhancing its signal recording efficiency [8,9,10]. Though various aspects and designs of the NW FETs have been explored, the fundamental mechanism behind the recording of the NW FETs and outperforming recording capability of the same is not quite yet studied.

Most of the reported FETs for neural recording operate as small voltage signal amplifier (e.g., common-source amplifier). However, because of the interface with electrolyte and cells, the overall circuitry of the recording amplifier system would be more complicated than just a simple electrical ac voltage amplifier. Since live cells are the signal source through the ionic movement across the cell membrane resulting in tens of mV range of transmembrane potential change and thus typically hundreds of µV range of extracellular potential change, how the cell-electrolyte-electrode interfaces are formed is very critical. In addition, purely electrical performance of the amplifiers such as gain, noise, and signal-to-noise ratio (SNR) must be carefully characterized to understand the mechanism and contribution of each process in overall recording system. However, most of reported works have neither fully provided aforementioned electrical characteristics of the amplifiers, nor separated individual contributions. With the absence of information, it is difficult to analyze and compare a newly designed system with others including both active and passive MEA systems. The usage of actual live cells did indeed make it hard to analyze each component and compare with passive MEAs. Although the ultimate goal of the systems is to directly communicate with live cells, it is impossible to utilize cells as reliable and controllable signal sources for the new recording system development and analysis: (1) cells of different kinds often generate significantly different signal intensities (e.g., cardiac cell signals are often an order of magnitude higher than neuronal cell signals) [9,10,11,12]; (2) the timing and waveform of the cell signals are random and inconsistent; (3) physical behavior of the cell(s)/electrolyte/electrode is also very difficult to control. Although direct comparison of the performance of the NW FET-based active MEAs with that of passive MEAs is mandatory, it was never conducted. 

In this work, we report in-depth electrical and electrochemical characterization of silicon NW FET-based active-type MEA designed for neural recording using precisely controlled artificial neuronal cell-like signals. Our silicon nanowire (SiNW) FET-based MEA (‘active-type MEA’) was configured to use an extended polysilicon gate microelectrode (‘extended-gate-type’) for better chemical stability and lower signal loss than other FET structures as described in the next section [13,14]. The performance of the active-type MEA was directly compared with that of platinum black (PtBk) microelectrode-based passive-type MEA. Platinum black electrodes are the most common type of recording electrodes used in neurophysiology. Detailed characterization and discussion of the noise of SiNW FETs are also presented.

## 2. Experimental Setup

**Figure 1:**
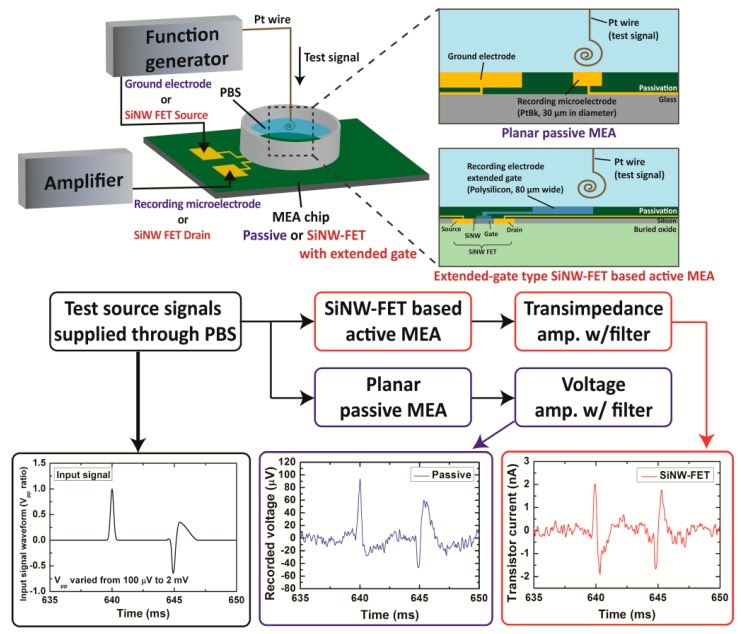
Overall measurement setup diagram.

Experimental setup in this work is divided by SiNW FET-based active MEA and PtBk passive MEA. The SiNW FETs are accompanied with a custom-built transimpedance amplifier (TIA) that amplifies and converts the drain currents of the transistors to output voltage signals. The amplified output voltage signals from both MEAs are simultaneously delivered to a PC through 24-bit analog-to-digital converter (ADC), NI 9239 from National Instrument Inc. (Austin, TX, USA), for analysis and side-by-side comparison. See the overall measurement system setup in Figure 1.

### 2.1. Silicon Nanowire Field-Effect Transistors (SiNW FETs)

**Figure 2:**
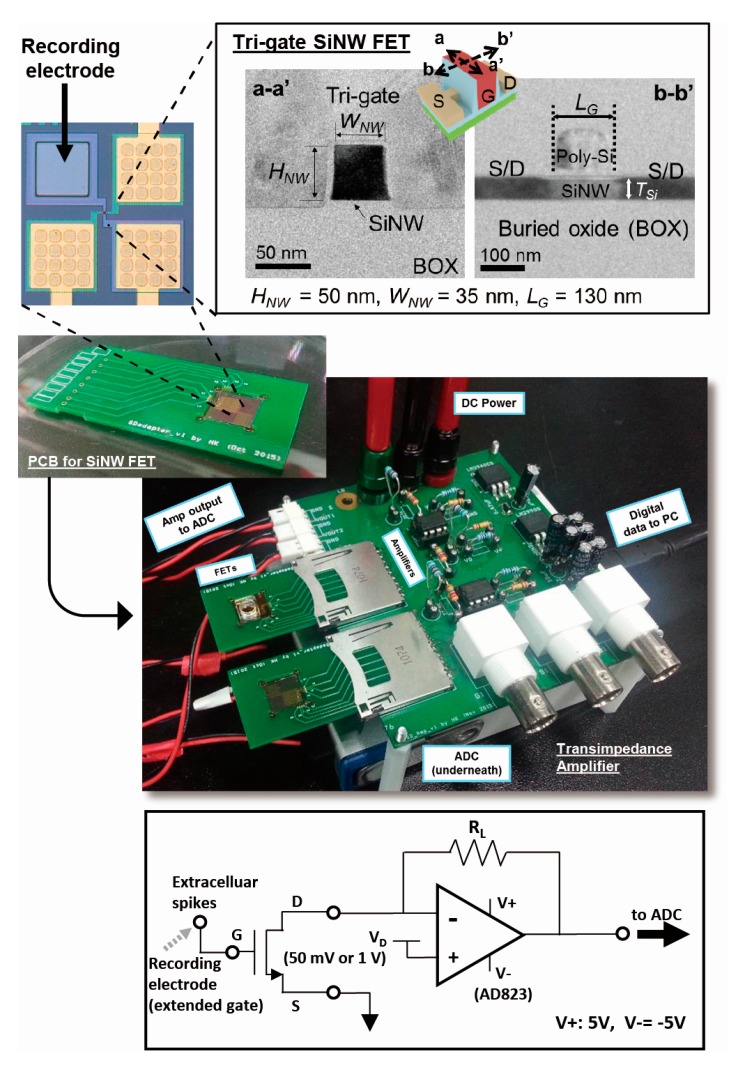
Silicon nanowire field-effect transistor and the transimpedance amplifier for the field-effect transistor FETs: Cross-sectional TEM image of the silicone nanowire (SiNW) FETs (**top right**); Top view of a SiNW FET and 80-µm extended recording electrode (**top left**); printed circuit board (PCB) for the SiNW FET wafer and the amplifier system (**middle**); circuit configuration for the transimpedance amplifier (**bottom**).

Various device designs of bulk silicon MOSFETs for neural recording were suggested. Common structures are OSFET (conventional bulk MOSFET without metal gate electrode) and FG-MOSFETs (MOSFETs with a floating gate for sensing) [1,15]. While OSFET structure could avoid the possibility of signal loss (e.g., capacitive division), exposing the thin gate dielectric to electrolyte for long time could physically damage the dielectric and significantly degrade the device performance. On the other hand, FG-MOSFET structure not only requires additional processing steps for the fabrication of the floating gate, but also always suffers capacitive division loss of the recording signal intensity [16]. Therefore, in this work, arrays of SiNW FETs with an extended polysilicon recording gate electrode (80-µm square shape) were chosen (200-µm center-to-center electrode spacing, 25 FETs/mm^2^) (see Figure 2). With this design, it is possible to protect the channel area of the transistors without any signal attenuation.

SiNW FETs was fabricated on a p-type silicon-on-insulator (SOI) wafer [17]. The thickness of top silicon was reduced to 50 nm by iterative thermal oxidation and diluted HF dipping. SiNWs were patterned by KrF scanner, and partial photoresist ashing [18] was followed to reduce the width of SiNWs from 180 nm to 50 nm. Then, a 3.6-nm thick SiO_2_ layer was formed by thermal oxidation for gate dielectric, and 100-nm thick n^+^ in-situ poly-Si layer was deposited by low-pressure chemical vapor deposition (LPCVD). After gate patterning, source and drain sides were doped with arsenic, and the dopants were activated by a rapid thermal annealing at 1000 °C for 5 s. The channel length of the SiNW FETs chosen for this work is 130 nm, and the channel width is ranged from 50 to 78 nm.

**Figure 3:**
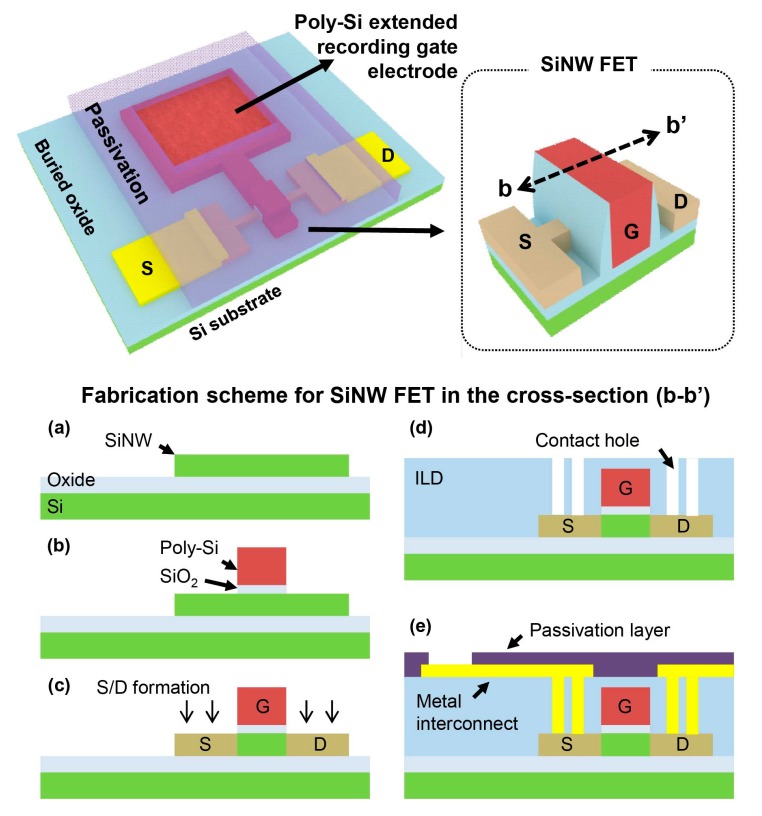
Fabrication scheme for the SiNW FET with an extended polysilicon recording gate electrode (**a**) silicon nanowire formation on SOI wafer; (**b**) SiO_2_ gate dielectric and polysilicon gate electrode formation; (**c**) source and drain electrodes formation by ion implantation; (**d**) SiO_2_ inter-layer dielectric deposition and contact hole formation; (**e**) Cr/Au interconnect formation, SU-8 passivation and contact pads opening.

For the back-end process, SiO_2_, Cr/Au and SU-8 layer was used for an inter-layer dielectric (ILD), interconnects and a passivation layer, respectively. By LPCVD process, 200-nm thick SiO_2_ for ILD was deposited, and then contact hole patterning and etching was followed. Afterward, forming gas annealing was carried out with ambient condition of 10% H_2_, and 90% N_2_ ambient at 400 °C for 30 min to reduce non-desired traps in SiNW FETs. Cr (10 nm)/Au (200 nm) for interconnects were sequentially deposited by an evaporator, and Cr/Au layer were patterned by a lift-off process. The recording electrode (80-μm rectangular shape) was exposed by etching the oxide layer on the extended poly-Si gate with 10:1 diluted HF. Finally, all devices except recording electrode were passivated by 2-μm thick SU-8 layer. Overall fabrication process flow is provided in Figure 3.

**Figure 4:**
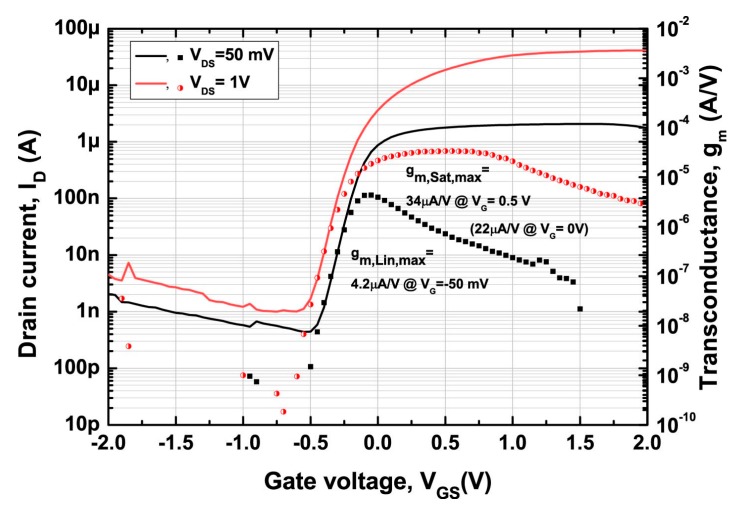
Transfer characteristic of a representative SiNW FET used in this study (solid lines). Extracted transconductance (dots) is also plotted and labeled as the 2nd y-axis on the right. Depletion mode characteristic is observed.

Transfer characteristic of the SiNW FETs are shown in Figure 4 Transconductance (*g_m_*), defined as the derivative of drain current with respect to gate voltage (∂*i_ds_/*∂*v_gs_*), is calculated from the *I_D_*-*V_GS_*. The transistors are depletion mode maximizing the *g_m_* at zero electrolyte dc bias (i.e., *V_GS_* = 0 V) while minimizing risk of electrochemical corrosion and damage [16]. The maximum *g_m_* of a representative transistor is 4.2 µA/V in linear mode (*V_DS_* = 50 mV, *V_GS_* = 50 mV), and 34 µA/V in saturation mode (*V_DS_* = 1 V, *V_GS_* = 0.5 V), respectively. Other important device parameters are summarized in Table 1. 

The fabricated SiNW FET wafer was diced to 1 cm × 1 cm size, wire-bonded to a SD-card shaped printed circuit board (PCB), and connected to a custom built low-noise TIA through a SD card socket (see Figure 2).

### 2.2. Low-Noise Transimpedance Amplifier (TIA) for SiNW FETs

The TIA was designed with a low-noise operational amplifier (AD823, Analog Devices, Inc., Norwood, MA, USA) as in Figure 2. 10: 32 AM he drain dc bias of the transistor is applied to the non-inverting input terminal such that non-inverting amplifier scheme was implemented while supplying reliable dc bias to the transistor. A feedback resistor was chosen appropriately to maximize the output ac voltage signal while minimizing the thermal noise of the resistor at the output node (only 20–45 pA of RMS noise current; i.e., less than 5 µV RMS input referred noise). The output voltage signal of TIA was digitally band-pass filtered (150 Hz–5.5 kHz) after the data acquisition by the ADC.

### 2.3. Passive MEA System 

A conventional microfabricated planar-type 60-channel passive MEA chip was fabricated and used with a low-noise voltage amplifier (gain: 1000 V/V). The chip was fabricated on a glass wafer using a 200-nm thick gold electrode layer with 20-nm thick titanium as adhesion layer and a 500-nm thick plasma-enhanced chemical vapor deposited silicon nitride as passivation insulator. The circular gold electrodes with a range of diameter from 30 µm to 90 µm (with 200-µm center-to-center electrode spacing; 25 electrodes/mm^2^) were electroplated with PtBk to reduce the impedance and thus noise of the electrodes. Thirty-micrometer electrodes were used for the data presented in this work, and larger electrodes (e.g., 90 µm) exhibited the same recording performance and drew the same conclusion of this study. The fabricated glass die was wire-bonded and packaged with a printed circuit board to interface with the signal amplification and recording system. Detailed structure and design of the passive MEA can be also found in [19]. The baseline RMS noise of the overall system was less than 10 µVrms. The output signal of the passive MEA was acquired and band-pass filtered in the same way as for the active MEA with 25 kHz sampling frequency.

### 2.4. Artificial Neuron Signal Measurement Setup

For the controlled test input signal, a series of Gaussian and extracellular neural spike signals were generated by a low noise function generator, Keysight 33500B (Keysight Technologies, Inc., Santa Rosa, CA, USA) as shown in Figure 1. The pulse duration was 3 ms. The extracellular spike waveform was reproduced from the actual extracellular neuronal spike recorded from cultured hippocampal neurons. The generated artificial neuron signal with varying amplitude with respect to the reference voltage (from hundreds of µV to a few mV) was supplied through a platinum wire. The Pt wire was submerged in 1× phosphate buffered saline (PBS) (purchased from Gibco™, Waltham, MA, USA), and the wire was placed as close to the recording electrodes as possible. As the recording reference electrode, we chose either an additional large gold electrode on the chip for the passive MEA, and the source terminals of the SiNW FETs or a large Pt wire dipped in PBS for the SiNW FET active MEA. Though the source electrodes of the FETs were passivated from the PBS, the highly insulating SU-8 passivation layer holds the entire input signal intensity between the potential near the recording electrode and the reference electrode, allowing precise and robust control of the recorded input signal voltage near the FETs.

## 3. Results

In this section, first, the performance of SiNW FET-based active MEA is characterized in comparison with that of the passive MEA. Second, the intrinsic noise of the active MEA and what it means to the neural recording will be discussed in detail.

### 3.1. Spike Signal Recording Measurement

**Figure 5:**
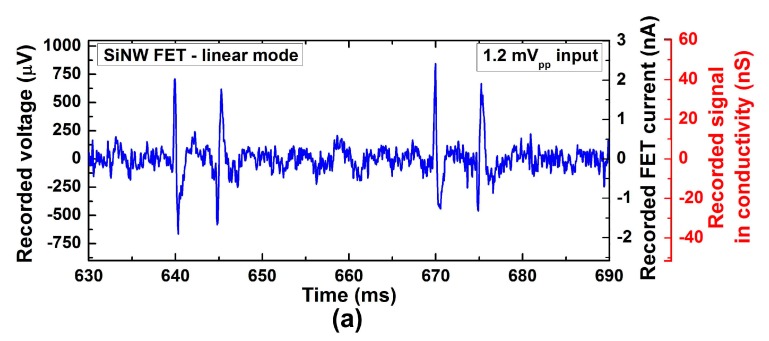

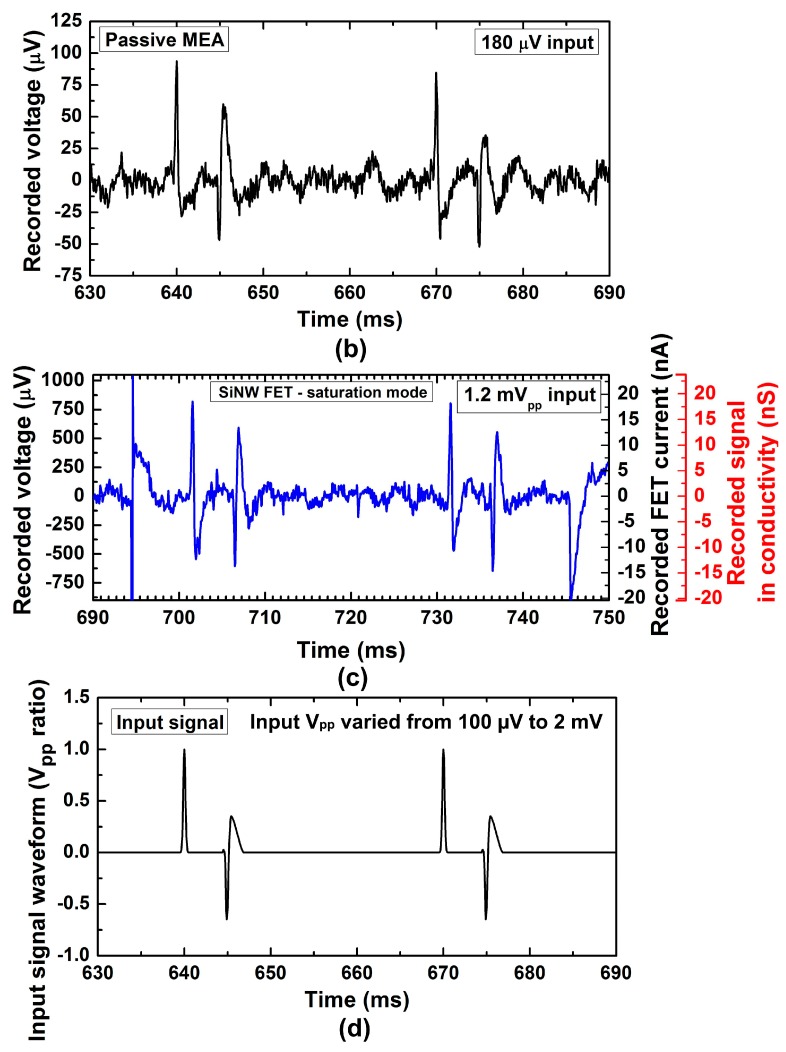
Recorded signals by (**a**,**c**) SiNW FET with 1.2 mV_pp_ input signal, and (**b**) passive microelectrode array (MEA) with 180 µV_pp_ input signal. Input signal waveform can be found in (**d**). SiNW FET operated (**a**) in linear mode (*V_DS_* = 50 mV), and (**c**) in saturation mode (*V_DS_* = 1 V). The amplified output signals were processed to recover the original signal at the recording electrode using the gain of amplifiers and transconductance of the FET. Recorded transistor current and conductance (*i_ds_/V_DS_*) corresponding to the data on the left y-axis before processing are labeled on the right y-axis. From the passive MEA, about 33% of signal strength reduction is observed due to the voltage drop by current flow from the signal Pt wire to on-chip ground reference electrode through PBS (from 180 µV_pp_ to 120 µV_pp_). Note that the two signal-like waveforms on both left and right ends in (**c**), which are not time-aligned with the input signals, are indeed artefacts generated from the FET intrinsic noise, which will be discussed in the next section.

Figure 5 shows representative recorded signals by both passive and active MEAs. Both MEAs showed nearly the same waveform as the input voltage signal. It indicates that there is no or negligible capacitive effect at both electrode–electrolyte interfaces due to double layer capacitance. The amplitude of recorded signals by SiNW FET was similar to what was theoretically estimated by the common source amplifier gain, *g_m_*. Depending on the operation mode of the transistors (i.e., either linear or saturation mode), the output current for 1.2-mV spike signals is 3.28 ± 0.09 nA in linear mode and 26.16 ± 0.52 nA in saturation mode operation. It refers to the output current/input voltage values of 2.74 ± 0.07 µA/V and 21.8 ± 0.43 µA/V, respectively, which are close to the extracted *g_m_* of 3.9 µA/V and 21.6 µA/V at zero gate bias in Figure 4. It is, therefore, theoretically possible to achieve nA-scale signal even from 100-µV spike signals (e.g., 100 µV × 21.6 µA/V results in 2.16 nA).

Unlike the passive MEA, SiNW FET-based MEA had difficulty in recording signal smaller than 1.2 mV due to its relatively high intrinsic RMS noise. SNR, defined as the peak-to-peak voltage of the recorded signal divided by RMS noise, was 4.7 ± 0.1 for linear and 4.1 ± 0.1 for saturation mode. In order to reliably detect spikes, SNR of five or larger is often used in threshold detection method where SNR is defined as zero-to-peak voltage divided by RMS noise [20,21,22]. Therefore, it is fair to say that 1.2 mV is the smallest signal to be recorded by our SiNW FETs. On the other hand, the passive MEA composed of platinized microelectrodes was able to record even 180-µV spike signals with much higher SNR (>9) as shown in Figure 5b. SiNW FETs are therefore outperformed by passive MEA in terms of SNR. Previously published SiNW FETs for various cell recordings reported their recording performance as sensitivity, defined as *g_m_*/*V_DS_* (ranged from 7 nS/mV to 32 nS/mV) [9,10,23,24]. The sensitivity of our SiNW FETs was 84 nS/mV in linear mode. Thus, to the best of our knowledge, our SiNW FETs were still much more powerful small signal amplifier than previously reported SiNW FETs.

One thing to note is that in 50-mV linear mode, the smallest signal to detect was almost identical to that in saturation mode despite a factor of eight difference in *g_m_*. It is because the intrinsic noise level of the FET decreases as the drain current decreases.

### 3.2. Intrinsic Noise of SiNW FETs 

**Figure 6:**
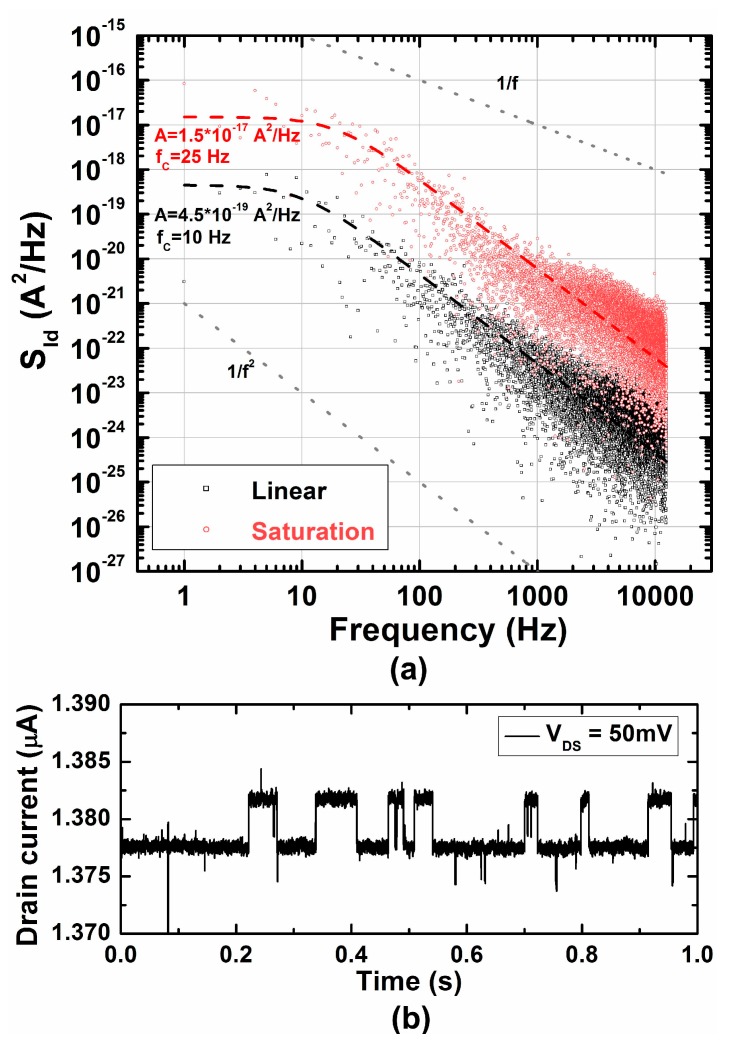

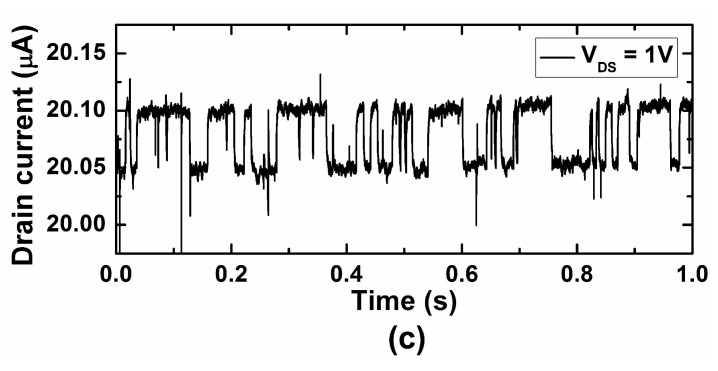
Noise characteristics of the SiNW FETs running as MEA. (**a**) Power spectral density (S_ID_) of drain current noise before band-pass filtering. The drain currents that represent random telegraph noise in time domain are given in (**b**) for linear mode, and in (**c**) for saturation mode.

As we observed in the previous section, the intrinsic noise of the FETs was found to be very important. Thus, the intrinsic noise of the SiNW FETs was characterized in detail. The intrinsic noise of the passive MEAs is mainly thermal noise from the impedance of the metal microelectrodes. The spectrum of the thermal noise is flat within the frequency range of our interest. The reduction of the electrode impedance is, therefore, of significant interest. On the other hand, active devices such as transistors show non-flat noise spectrum with monotonic decrease of its power spectral density with increasing frequency. The lowest RMS drain current noise of the SiNW FETs was 1.15 nA before band-pass filter and 475 pA after filtering. At 1 kHz, the drain current power spectral density (*S_Id_*) in linear mode was around 10^−22^ A^2^/Hz, which corresponds to 9.44 pA/Hz^0.5^ as shown in Figure 6a. It sits within the range of what have been reported for the 1/f noise of SiNW FETs [25,26]. It suggests that there is a room for improvement for the intrinsic noise of the SiNW FETs used in this work.

While some of the transistors showed typical 1/*f* characteristics (not shown here), some other transistors used in this study showed typical Lorentzian spectrum which is flat below a corner frequency (*f_c_*) and drops with 1/f^2^ shape as described in Equation (1) and shown in Figure 6a [27].

(1) $$S_{Lorentzian}\left(f\right)=\frac{A}{1+{\left(\frac{f}{f_{c}}\right)}^{2}}$$

where *A* is constant in A^2^/Hz as specified in Figure 6a. This behavior is often observed in nanoscale transistors and known as random telegraph noise (RTN) due to its transient response which resembles a telegraph signal. In Figure 6b,c, the time domain characteristics of RTN corresponding to the spectra in Figure 6a are shown. The *f_c_* in saturation was slightly higher than in linear, showing more frequent telegraph signal change. The RMS noise was 2.32 nA before band-pass filter and 476 pA after filtering.

**Figure 7:**
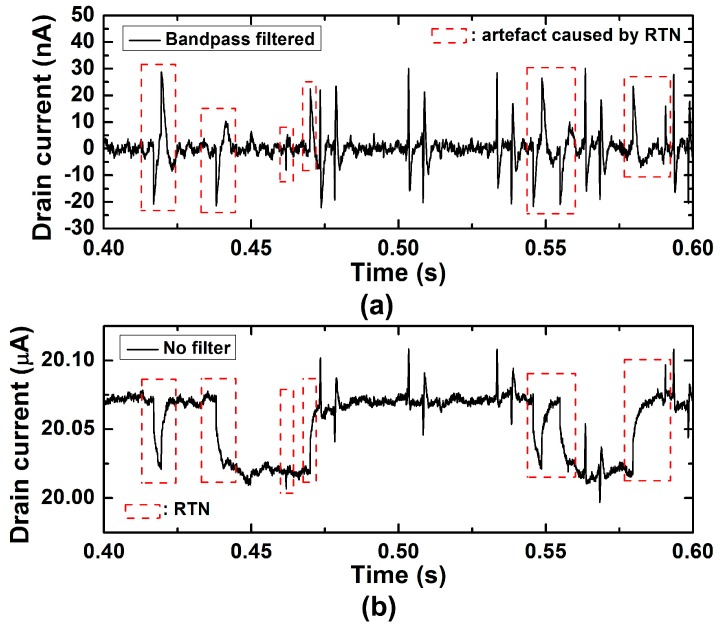
Artefacts caused by random telegraph noise. (**a**) Recorded signal after band-pass filter. Red dashed rectangle areas represent the artefacts caused by the edges of RTN in (**b**). (**b**) Recorded signal before band-pass filter. Red dashed rectangle areas represent the edges of RTN that create artefacts in (**a**).

Although the band-pass filter helped to reduce the RMS noise level, band-passed RTN created detrimental artefacts: because the RTN consists of a series of square waves, the abrupt change of both falling and rising edges of square waves after the band-pass filtering resembles neural spike signals as shown in Figure 7. The artefacts were, however, slower than typical neural spikes. As a benefit of using precisely controlled input signal, we were able to separate the actual recorded signals from the artefacts by RTN.

## 4. Discussion

**Figure 8:**
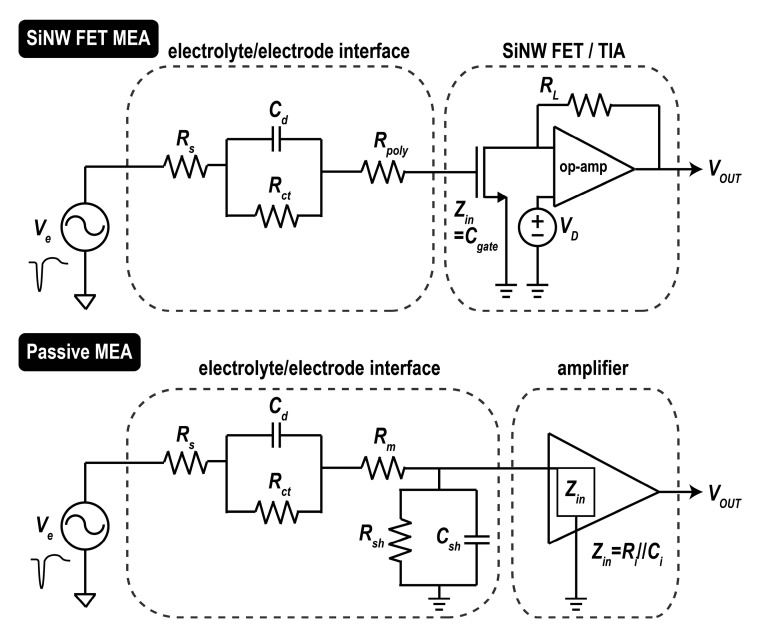
Equivalent circuits of overall extracellular potential recording MEA systems. *V_e_*: extracellular potential, *R_S_*: solution resistance, *C_d_*: double-layer capacitance, *R_ct_*: charge-transfer resistance, *R_m_*: metal interconnect resistance, *R_poly_*: poly-Si electrode resistance, *R_sh_* and *C_sh_*: shunt resistance and capacitance for the amplifier, *Z_in_*: input impedance of the amplifier and SiNW FET, *R_L_*: feedback resistance.

Equivalent circuits of overall MEA systems are drawn in Figure 8. The source voltage signal is connected to high input impedance amplifiers: for the active MEA, the gate terminal of SiNW FETs; and for the passive MEA, an input terminal of operational amplifier, AD823. In between, the resistance of the buffer solution, electrode-electrolyte double layer capacitor interface and resistance of recording electrodes (poly-Si, gold wire, respectively) are connected in series. The amplitude of the recorded signal is the small signal voltage at the input node of both MEAs. Depending on the values of the components in the circuit, the recorded voltage may experience voltage division from the source voltage (*V_e_*). Therefore, higher input impedance of the amplifiers minimizes the signal loss. The input impedance of the SiNW FET is dominated by the small gate capacitance. For the thickness of oxide (*t_ox_*) of 5 nm, 130 nm × 50 nm device area (*L* × *W*) leads to only 45 aF. Assuming the gate dielectric has good insulation quality, the input impedance of the active MEA would be even higher than 1 TΩ at 2 kHz. On the other hand, the input impedance of op amp in non-inverting amplifier configuration is roughly composed of an input capacitor (*C_i_*) and an input resistor (*R_i_*) in parallel. For AD823 which is a JFET-input-based op amp, the input impedance is 10 TΩ//1.8 pF, and it is 44 MΩ at 2 kHz. Although experimental confirmation is needed, it could be expected that the SiNW FETs with higher input impedance would record actual cellular signals through the electrolyte–electrode interface more efficiently than the passive MEA when *R_s_* in Figure 8 is increased significantly due to any biological reason (i.e., highly resistive path is formed between the cell and the electrode). For example, the glial scar formation—which is the most common implant failure—around the MEA recording electrodes after implantation could increase the *R_s_*.

However, when the path is rarely resistive as in this study, SNR of SiNW FET-based recording was much poorer than the planar passive MEA. Despite the usage of 6.7 times stronger input signal, the SNR was nearly 50% smaller. Thus, planar passive MEA outperforms the SiNW FETs with more than an order of magnitude higher SNR for the same strength input signal. Significantly high intrinsic noise of the SiNW FETs was found to be the reason.

In order to record and analyze neural signal better, therefore, the key factors are: (1) how efficiently the source signal is transferred to the amplifier; (2) how strongly the amplifier can amplify the signal; and (3) how low the intrinsic noise can be minimized. In addition, how closely cells make contact to the recording electrodes such as cell/nanostructure can also affect the recording capability. In this study, the amplifier part of SiNW FET MEA was carefully characterized. It is concluded that the electrically confirmed common source amplifier characteristic, *g_m_*, is maintained in neural recording setup.

The message from this section is the following: in theory, SiNW-FET-based active MEAs with extended recording electrode can convert extracellular potentials (hundreds of µV) to significantly large current signals (nA range), but the intrinsic transistor noise is the limiting factor for achieving good SNR. So, Lieber and others work that showed high quality recordings were probably due to the cell-electrode coupling effect, which generated even larger input signal, overpowering the intrinsic noises [8,9,10]. This is often not possible in passive MEA as the electrode is comparable to the cell size (µm scale). Recent work by Spira supports our hypothesis of larger signals due to good cell-electrode coupling [28]. With our results in this work, we can experimentally confirm that SiNW FET-based active MEA recording will be only beneficial if the ‘right’ coupling effect occurs. Or, the coupling effects may occur more readily in nano-size electrodes.

Although the 1/f noise level of the SiNW FETs in this work was reasonable (9.44 pA/Hz^0.5^ at 1 kHz), the intrinsic noise of FETs was found to be limiting smaller signal recording. Based on the observation of RTN, the carrier number fluctuation is the dominant origin of 1/f noise in our SiNW FETs. Therefore, the power spectral density of the drain current noise (*S_id_*) is proportional to the width (W) of the MOSFETs [29,30].

(2) $$S_{Id}\left(f\right)\propto \frac{kTI_{d}^{2}}{fWL^{2}},$$

(3) $$I_{D,lin}\propto g_{m}\propto W,$$

(4) $$S_{Id}\left(f\right)\propto W,$$

Because the RMS noise is proportional to *S_Id_*^0.5^, the intrinsic noise of FETs is proportional *W*^0.5^. Because the transconductance, *g_m_*, is proportional to *W*, the recorded signal would be proportional to *W*. Therefore, SNR (∝*I_d_*/*S_Id_*^0.5^) in MOSFETs is theoretically proportional to *W*^0.5^. SiNW FETs used in this work has extremely small width, as large as only 78 nm. Therefore, increasing only the width of the SiNW FETs will be able to simply improve SNR, enabling much smaller signal recording. For example, increasing *W* to 1 µm could improve the SNR more than a factor of four. Thus, 300-µV signals would be detectable with good SNR. Even after doing so, the SiNW FETs are still much smaller than the passive MEA electrodes (i.e., 30 µm).

## 5. Conclusions

In this study, we extensively tested the performance, characteristics, and fundamentals of SiNW FET-based active-type MEA for neural recording and compared with more popularly used PtBk microelectrode-based passive-type MEA. Rather than merely focusing on the demonstration of cell signal recording without deeply understanding the mechanism of the recording process, we carefully analyzed and understood all the characteristics of the SiNW FETs as a front-end neural signal sensing or amplifying device. The following are key conclusions from the study. The intensity of the recorded extracellular spikes by SiNW FETs is exactly determined by the transconductance of the transistors. This means that the electrical characteristics of the transistors directly tell us how well the active MEA can perform. In our SiNW FETs, 1.2-mV signal is found to be the smallest detectable signal with the SNR of about five. While 1.2-mV may be suitable for cardiac cell recording [11,12], smaller signal recording capability of the FETs was poorer than the passive MEA due to the high intrinsic noise of the FETs. If the actual cell recording results using similar FETs outperform the transconductance, then there may be additional contributions to the recording, such as good cell-electrode coupling, that leads to much higher extracellular potentials (mV range) closer to tens of mV transmembrane potentials. 

For further SNR improvement, 1/f noise needs to be reduced, and geometric optimization of the FETs will be able to enhance the SNR based on the 1/f noise model discussed in this work. In addition to the typical 1/f noise, RTN was observed in some of our SiNW FETs. It was observed that the RTN often observed in nanoscale transistors causes artefacts which could look like slow extracellular spikes and make spike sorting inaccurate. Designing wider FETs would alleviate both issues by reducing relative 1/f noise and chance of RTN. We should also consider developing a post-processing algorithm to filter out the artefacts possibly by comparing both filtered and unfiltered data. While the spike signals are unchanged after bandpass filtering, the artefacts caused by RTN before and after bandpass filter is clearly different. In addition, the artefact waveform often varies more slowly than typical extracellular spikes (three milliseconds).

Many different transistor technologies have been suggested and demonstrated for cell signal recording. However, usage of different cells at different development stages, different designs of the transistors, and different environments make it difficult to compare their performances. The two systems we studied in this work may not promise that they can represent all kinds of previously reported systems. However, we believe the transistor device knowledge developed and tested in this work can help more in-depth understanding of the neural recordings using transistors, and provide a solid design guideline for new types of FET developments and improvements in neural recording including our own SiNW FETs (e.g., noise improvement through better gate oxide or novel device structure).

## Figures and Tables

**Table 1:** Device Parameters for SiNW FETs.

Symbol	Quantity	Value
L	Transistor length	130 nm
W	Transistor width	50–78 nm
*t_ox_*	Gate oxide thickness	5 nm
*C_ox_*	Areal gate capacitance	0.69 µF/cm^2^
*C_ox_* × LW	Gate capacitance	45–70 aF
*t_Si_*	Thickness of Si body	50 nm
SS	Subthreshold swing	71 mV/dec
DIBL	Drain-induced barrier lowering	0.04 V/V
*V_T_*	Threshold voltage	−0.18 V
